# ﻿A new species of *Hemiphyllodactylus* (Squamata, Gekkonidae) from Ha Giang Province, Vietnam

**DOI:** 10.3897/zookeys.1167.103713

**Published:** 2023-06-22

**Authors:** Vinh Quang Luu, Thuong Huyen Nguyen, Quyen Hanh Do, Cuong The Pham, Tuoi Thi Hoang, Truong Quang Nguyen, Minh Duc Le, Thomas Ziegler, Jesse L. Grismer, L. Lee Grismer

**Affiliations:** 1 Faculty of Forest Resources and Environmental Management, Vietnam National University of Forestry, Xuan Mai, Chuong My, Hanoi, Vietnam; 2 Herpetology Laboratory, Department of Biology, La Sierra University, 4500 Riverwalk Parkway, Riverside, California 92505, USA; 3 Faculty of Environmental Sciences, University of Science, Vietnam National University, Hanoi, 334 Nguyen Trai Road, Hanoi, Vietnam; 4 Institute of Ecology and Biological Resources, Vietnam Academy of Science and Technology, 18 Hoang Quoc Viet Road, Hanoi, Vietnam; 5 Graduate University of Science and Technology, Vietnam Academy of Science and Technology, 18 Hoang Quoc Viet Road, Hanoi, Vietnam; 6 Central Institute for Natural Resources and Environmental Studies, Vietnam National University, Hanoi, 19 Le Thanh Tong, Hanoi, Vietnam; 7 Department of Herpetology, American Museum of Natural History, Central Park West at 79; 8 th; 9 Street, New York, New York 10024, USA; 10 AG Zoologischer Garten Köln, Riehler Strasse 173, D-50735 Cologne, Germany; 11 Department of Herpetology, San Diego Natural History Museum, PO Box 121390, San Diego, California, 92112, USA

**Keywords:** Genetics, *Hemiphyllodactyluslungcuensis* sp. nov., integrative approach, karst forest, morphology, Southeast Asia

## Abstract

An integrative analysis recovered a new species of the *Hemiphyllodactylustypus* group from a karst formation in Lung Cu Commune, Dong Van District, Ha Giang Province, northeastern Vietnam. *Hemiphyllodactyluslungcuensis***sp. nov.** is embedded within clade 6 of the *typus* group, bearing an uncorrected pairwise sequence divergence of 4.6–20.2% from all other species based on a 1,038 base pair segment of the mitochondrial NADH dehydrogenase subunit 2 gene (ND2). It is diagnosable from other species in clade 6 by statistically significant mean differences in normalized morphometric, meristic, and categorical characters. A multiple factor analysis using the three aforementioned character types recovered its unique, non-overlapping placement in morphospace as statistically significantly different from that of all other species in clade 6. The description of this new *Hemiphyllodactylus* species contributes to a growing body of literature underscoring the high degree of herpetological diversity and endemism in karst landscapes in Vietnam as well as in the genus *Hemiphyllodactylus*.

## ﻿Introduction

**Figure 1.**
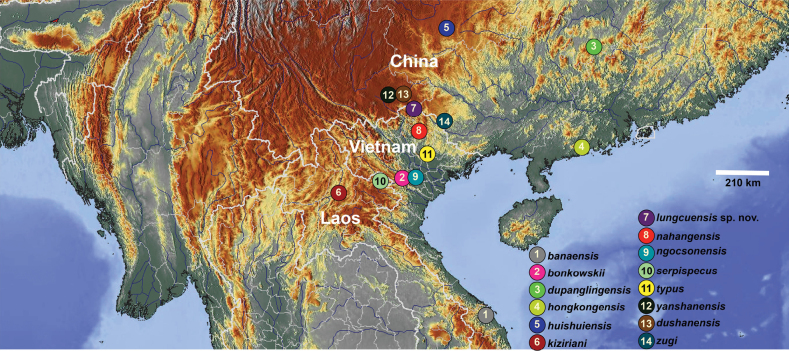
Distribution of the species of clade 6 of the genus *Hemiphyllodactylus*.

The gekkonid genus *Hemiphyllodactylus* Bleeker, 1860 consists of 54 species that are widely distributed from southern India and Sri Lanka, across Southeast Asia to the western Pacific (Zug 2010; Grismer et al. 2013, 2018; Agarwal et al. 2019; Agung et al. 2022; Uetz et al. 2022). Despite this broad distribution, their relatively small size, low densities, localized distributions, and cryptic coloration, render them inconspicuous components of the microenvironments they inhabit (Grismer et al. 2020). Bourret (1937) described the first species of *Hemiphyllodactylus* from Vietnam, namely *H.typuschapaensis* from Sa Pa, Lao Cai Province. Zug (2010), based on the examination of the holotype of *H.typuschapaensis*, considered it to be referable to *H.yunnanensis* Boulenger. Since then, a total of seven species of *Hemiphyllodactylus* have been recognized in the country, including *H.yunnanensis* Boulenger, 1903 from northern regions (Zug 2010); *H.zugi* Nguyen, Lehmann, Le, Duong, Bonkowski and Ziegler 2013 from Cao Bang Province; *H.banaensis* Ngo, Grismer, Pham and Wood 2014 from Da Nang City; *H.bonkowskii* and *H.ngocsonensis* Nguyen, Do, Ngo, Pham, Pham, Le and Ziegler 2020 from Hoa Binh Province; *H.nahangensis* Do, Pham, Phan, Le, Ziegler and Nguyen 2020 from Tuyen Quang Province; and *H.dalatensis* Do, Nguyen, Le, Pham, Ziegler and Nguyen 2021 from Lam Dong Province (Fig. 1).

Based on phylogenetic analyses conducted in previous studies, *Hemiphyllodactylus* species belonging to clade 6 of the *Hemiphyllodactylustypus* group have been designated (Agung et al. 2021, 2022). This group includes various species such as *H.banaensis*, *H.bonkowskii*, *H.nahangensis*, *H.ngocsonensis*, and *H.zugi* from Vietnam; *H.kiziriani* Nguyen, Botov, Le, Nophaseud, Zug, Bonkowski, and Ziegler 2014, *H.serpispecus* Eliades, Phimmachak, Sivongxay, Siler and Stuart 2019 from Laos; and *H.dupanglingensis* Zhang, Qian, Jiang, Cai, Deng and Yang 2020, *H.dushanensis* Zhou, Liu and Yang 1981, *H.hongkongensis* Sung, Lee, Ng, Zhang and Yang 2018, *H.huishuiensis* Yan, Lin, Guo, Li and Zhou 2016; *H.yanshanensis* Agung, Chornelia, Grismer, Grismer, Quah, Lu, Tomlinson and Hughes 2022 from southern China.

During herpetological surveys in Lung Cu Commune, Dong Van District, Ha Giang Province, northeastern Vietnam, we collected seven specimens of an unnamed species of *Hemiphyllodactylus*. The new population differed from the remaining known species of the genus based on morphological and molecular phylogenetic analyses. Therefore, we herein describe it as a new species.

## ﻿Materials and methods

### ﻿Species delimitation

The general lineage concept (GLC: de Queiroz 2007) adopted herein proposes that a species constitutes a population of organisms evolving independently from other such populations owing to a lack of, or limited gene flow. By “independently” it is meant that new mutations arising in one species cannot spread readily into another species (Barraclough et al. 2003; de Queiroz 2007). Molecular phylogenies recovered multiple monophyletic mitochondrial lineages of individuals (populations) that were used to develop initial species-level hypotheses, the grouping stage of Hillis (2019). Discrete color pattern data and univariate and multivariate analyses of morphological data were then used to search for characters and morphospatial patterns consistent with the tree-designated species-level hypotheses, the construction of boundaries representing the hypothesis-testing step of Hillis (2019), thus providing independent diagnoses to complement the molecular analyses. In this way, delimiting (phylogeny) and diagnosing (taxonomy) species are not conflated (Frost and Hillis 1990; Frost and Kluge 1994; Hillis 2019).

### ﻿Sampling

Field surveys were conducted during the month of August in 2017 and 2022 in Lung Cu Commune, Dong Van District, Ha Giang Province, northeastern Vietnam. After having been photographed alive, newly collected individuals, were anaesthetized and euthanized in a closed vessel with a piece of cotton wool containing ethyl acetate (Simmons 2002), fixed in 85% ethanol, thereafter and subsequently transferred to 70% ethanol for permanent storage. Tissue samples were preserved separately in 70% ethanol. Specimens were deposited in the collection of the Vietnam National University of Forestry (VNUF) and the Institute of Ecology and Biological Resources (IEBR), Hanoi, Vietnam.

### ﻿Morphological data

Terminology of morphological characters follows Zug (2010), Nguyen et al. (2013), and Grismer et al. (2021). Measurements (morphometrics) were taken with a digital caliper to the nearest 0.1 mm on the left side of the body. Character abbreviations and descriptions are as follows: SVL: snout-vent length (from the tip of snout to the vent); TaL: tail length (from the vent to the tip of the tail, original and regenerated); TrunkL: trunk length (from the posterior margin of the forelimb at its insertion point on the body to the anterior margin of the hindlimb insertion point on the body); HeadL: head length (from the posterior margin of the retroarticular process of the lower jaw to the tip of the snout); HeadW: head width (measured at the angle of the jaws); EyeD: eye diameter (the greatest horizontal diameter of the eyeball); SnEye: snout-eye length (from anteriormost margin of the eyeball to the tip of snout); NarEye: nares-eye length (from the anterior margin of the eyeball to the posterior margin of the external nares); SnW: internarial width (measured between the nares across the rostrum); and EarD: ear diameter (maximum diameter of the ear opening).

The following meristic characters were counted using the same dissecting microscope: Chin: chin scales (the number of scales contacting the infralabials and mental from the juncture of the second and third infralabials on the left side to the juncture of the second and third infralabials on the right side); CN: circumnasal scales (the number of scales abutting the external naris, exclusive of the rostral and first supralabial); SnS: the number of scales between the supranasals; SL: supralabial scales (number of enlarged scales bordering the mouth on the upper jaw from the rostral to a point in line with the posterior margin of the orbit); IL: infralabial scales (the number of enlarged scales bordering the mouth on the lower jaw from the mental to a point in line with the posterior margin of the orbit); VS: ventral scales (the number of longitudinal ventral scales at mid-body contained within one eye diameter); DS: dorsal scales (the number of longitudinal dorsal scales at mid-body contained within one eye diameter); lamellae formulae determined as the number of U-shaped, subdigital lamellae (split and single) on the digital pads on digits II–V of the hands and feet (on the left); SL1F: the number of subdigital lamellae wider than long on the first finger; SL1T: the number of subdigital lamellae wider than long on the first toe; the total number of precloacal and femoral pores (i.e. the contiguous or discontinuous rows of femoral and precloacal pore-bearing scales); and CloacS: the number of cloacal spurs.

The categorical color pattern characters evaluated were the presence or absence of dark markings on the dorsum (BodPartn); presence or absence of a dark pre– and/ or postorbital stripe extending onto at least the neck (PosOStrp); the presence or absence of a linear series of white postorbital and dorsolateral spots on the trunk (PosOTrSpt); and the presence or absence of pale–colored, anteriorly projecting arms of the pale–colored postsacral marking (PosSacrlMakg).

### ﻿Genetic data

We obtained 1,038 base pairs of the NADH dehydrogenase subunit 2 (ND2) gene from 45 specimens from GenBank and five newly sequenced specimens from Lung Cu Commune, Dong Van District, Ha Giang Province, northeastern Vietnam for the phylogenetic analyses (Table 1). Catalog numbers are abbreviated as follows: **CSUFT**, Central South University of Forestry and Technology, Changsha, China; **ITBCZ**, Institute of Tropical Biology, Zoological Collection; **KIZ**: Kunming Institute of Zoology; **LSUHC**, La Sierra University Herpetology Collection; **NJUN**, Nanjing Normal University, Nanjing; **MVZ**, Museum of Vertebrate Zoology; **NUOL**, National University of Laos; **SYS**, The Museum of Biology, Sun Yatsen University (SYS), Guangzhou; **ZFMK**, Zoologisches Forschunginstitut und Museum Alexander Koenig, Bonn, Germany. *Hemiphyllodactylusharterti* (Werner, 1900) of the *H.harterti* group was used to root the tree following Grismer et al. (2013). For molecular phylogenetic analyses, we used the protocols of Le et al. (2006) for DNA extraction, amplification, and sequencing. Tissue samples were extracted using DNeasy blood and tissue kit, Qiagen (California, USA). Extracted DNA from the fresh tissue was amplified by PCR mastermix (Fermentas, Canada). A fragment of the mitochondrial gene ND2 (NADH dehydrogenase subunit 2) was amplified using the primer pair ND2f101A (CAACAGAAGCCACAACAAAAT) and HemiR (GAAGAAGAGGCTTGGKAGGCT) (Nguyen et al. 2013). The PCR volume consisted of 21 μl (10 μl of mastermix, 5 μl of water, 2 μl of each primer at 10 pmol/μl and 2 μl of DNA or higher depending on the quantity of DNA in the final extraction solution). The following temperature profile for PCR was used: 95 °C for 5 min to activate the taq; with 40 cycles at 95 °C for 30 s, 50 °C for 45 s, 72 °C for 60 s; and the final extension at 72 °C for 6 min. PCR products were subjected to electrophoresis through a 1% agarose gel (UltraPureTM, Invitrogen). Gels were stained for 10 minutes in 1×TBE buffer at 2 pg/ml of ethidium-bromide and visualized under UV light. Successful amplifications were purified to eliminate PCR components using GeneJETTM PCR Purification kit (Fermentas, Canada). Purified PCR products were sent to 1^st^ Base (Selangor, Malaysia) for sequencing.

**Table 1.** Samples used for the phylogenetic analyses.

Species	Catalog no.	Locality	GenBank no.
*Hemiphyllodactylusbanaensis*	ITBCZ 2450	Ba Na-Nui Chua, Vietnam	KF219783
*H.bonkowskii*	IEBR 4694	Hang Kia-Pa Co NR, Mai Chau, Hoa Binh, Vietnam	MT415553
	IEBR 4695	Hang Kia-Pa Co NR, Mai Chau, Hoa Binh, Vietnam	MT415554
*H.dupanglingensis*	CSUFT 00401	Dupangling, Hunan, China	MT576070
	CSUFT 00405	Dupangling, Hunan, China	MT576071
*H.hongkongensis*	SYS r001728	Aberdeen Country Park, Hong Kong	MF893330
	SYS r001729	Aberdeen Country Park, Hong Kong	MF893331
	SYS r001730	Aberdeen Country Park, Hong Kong	MF893332
	SYS r001735	Aberdeen Country Park, Hong Kong	MF893333
*H.huishuiensis*	NJNUh00736	Huishui, Guizhou, China	KU519708
	NJNUh00851	Huishui, Guizhou, China	KU519707
	NJNUh00852	Huishui, Guizhou, China	KU519709
	NJNUh00857	Huishui, Guizhou, China	KU519710
	NJNUh00858	Huishui, Guizhou, China	KU519711
*H.kiziriani*	IEBR A.2014.3	Luang Prabang, Laos	KJ676800
	IEBR A.2014.4	Luang Prabang, Laos	KJ676801
	IEBR A.2014.5	Luang Prabang, Laos	KJ676802
*Hemiphyllodactyluslungcuensis* sp. nov.	VNUF R.2021.01	Lung Cu, Dong Van, Ha Giang, Vietnam	OR067852
	IEBR R.5151	Lung Cu, Dong Van, Ha Giang, Vietnam	OR067853
	VNUF R.2021.03	Lung Cu, Dong Van, Ha Giang, Vietnam	OR067851
	VNUF R.2023.01	Lung Cu, Dong Van, Ha Giang, Vietnam	OR067854
	VNUF R.2023.02	Lung Cu, Dong Van, Ha Giang, Vietnam	OR067855
*H.nahangensis*	IEBR 4741	Na Hang, Tuyen Quang, Vietnam	MT711191
	IEBR 4742	Na Hang, Tuyen Quang, Vietnam	MT711192
	IEBR 4743	Na Hang, Tuyen Quang, Vietnam	MT711193
*H.ngocsonensis*	IEBR 4689	Ngoc Son-Ngo Luong NR, Lac Son, Hoa Binh, Vietnam	MT415551
	IEBR 4690	Ngoc Son-Ngo Luong NR, Lac Son, Hoa Binh, Vietnam	MT415552
*H.serpispecus*	NUOL 00476	Tham Ngou Leium Cave, Viengxay, Houaphan, Laos	MK307996
*H.typus*	MVZ 226500	Vinh Phuc, Vietnam	KF219798
*H.yanshanensis*	KIZ062101	Yunnan, China	ON676172
	KIZ062102	Yunnan, China	ON676173
	KIZ062090	Yunnan, China	ON676161
	KIZ062091	Yunnan, China	ON676162
	KIZ062092	Yunnan, China	ON676163
	KIZ062093	Yunnan, China	ON676164
	KIZ062094	Yunnan, China	ON676165
	KIZ062095	Yunnan, China	ON676166
	KIZ062096	Yunnan, China	ON676167
	KIZ062097	Yunnan, China	ON67618
	KIZ062098	Yunnan, China	ON676169
	KIZ062099	Yunnan, China	ON676170
	KIZ062100	Yunnan, China	ON676171
*H.dushanensis*	isolate_N1	Yunnan, China	FJ971016
	isolate_N2	Yunnan, China	FJ971017
*H.zugi*	ZFMK 94782	Ha Lang, Cao Bang, Vietnam	KF575153
	IEBR A.2013.20	Ha Lang, Cao Bang, Vietnam	KF575151
	IEBR A.2013.21	Ha Lang, Cao Bang, Vietnam	KF575152
*H.harterti*	LSUHC10383	Bukit Larut, Malaysia	KF219760
	LSUHC10384	Bukit Larut, Malaysia	KF219761

### ﻿Phylogenetic analyses

Maximum likelihood (ML) and Bayesian inference (BI) analyses were used to estimate the phylogenetic relationships among the sampled sequences in the alignment. An ML phylogeny was estimated using the IQ-TREE webserver (Nguyen et al. 2015; Trifinopoulos et al. 2016) preceded by the selection of substitution models using the Bayesian Information Criterion (BIC) in Model Finder (Kalyaanamoorthy et al. 2017), which supported TN+F+G4 as the best fit model of evolution for ND2 codon position 1, TN+F+G4 for position 2 and TIM2+F + G4 for position 3. One thousand bootstrap pseudoreplicates via the ultrafast bootstrap (UFB; Hoang et al. 2018) approximation algorithm were employed and nodes having ML UFB values of 95 and above were considered highly supported (Minh et al. 2013).

The Bayesian inference (BI) analysis was carried out using Mr Bayes 3.2.7. (Ronquist et al. 2012) XSEDE on the CIPRES Science Gateway (Cyberinfrastructure for Phylogenetic Research; Miller et al. 2010), employing the GTR+I+G model of evolution to all partitions. Two independent Markov chain Monte Carlo (MCMC) simulations were performed each with four chains, three hot and one cold. The MCMC simulation was run for 100 million generations, sampled every 10,000 generations, and the first 10% of each run was discarded as burn-in. Convergence and stationarity of all parameters from both runs were checked using Tracer v1.6 (Rambaut et al. 2014) to ensure effective sample sizes (ESS) were above 200. Post-burn-in sampled trees from both runs were combined using the sumt function in MrBayes and a 50% majority-rule consensus tree was constructed. Nodes with Bayesian posterior probabilities (BPP) of 0.95 and above were considered highly supported (Huelsenbeck et al. 2001; Wilcox et al. 2002). Uncorrected pairwise sequence divergences (p-distance) among and within species were calculated in MEGA11 (Kumar et al. 2016) (Table 2).

**Table 2.** Uncorrected genetic *P*-distances (%) in ND2 gene of *Hemiphyllodactylus*. Intraspecific *p*-distance are in bold font, n/a = data not applicable.

	*banaensis*	*bonkowskii*	*dupanglingensis*	*dushanensis*	*harterti*	*hongkongensis*	*huishuiensis*	*kiziriani*	*lungcuensis* sp. nov.	*nahangensis*	*ngocsonensis*	*serpispecus*	*typus*	*yanshanensis*	*zugi*
***banaensis***	**n/a**														
***bonkowskii***	13.0	**0.01**													
***dupanglingensis***	16.9	15.3	**0.00**												
***dushanensis***	17.3	14.7	5.1	**0.00**											
***harterti***	30.4	26.5	27.8	29.1	**0.04**										
***hongkongensis***	16.3	14.6	7.9	7.1	27.5	**0.01**									
***huishuiensis***	15.7	15.4	7.1	6.4	28.9	7.9	**0.01**								
***kiziriani***	22.7	20.0	20.3	20.5	29.6	20.0	20.5	**0.00**							
***lungcuensis* sp. nov.**	15.8	13.8	6.2	4.8	27.2	7.0	5.3	20.2	**0.02**						
***nahangensis***	15.5	13.6	5.8	5.5	25.3	7.8	6.3	20.1	4.6	**0.01**					
***ngocsonensis***	14.5	12.8	7.6	7.9	27.0	9.2	7.8	19.3	6.6	7.0	**0.02**				
***serpispecus***	12.2	7.5	16.6	16.0	25.7	16.4	15.4	21.4	14.4	13.2	12.6	**n/a**			
***typus***	15.2	14.6	7.2	6.3	27.9	8.9	7.4	21.2	5.6	4.1	8.8	15.6	**n/a**		
***yanshanensis***	17.1	15.2	8.6	8.3	26.0	9.4	4.4	22.2	6.8	7.3	8.5	14.7	8.2	**0.00**	
***zugi***	16.0	14.0	4.8	5.8	27.0	7.4	7.0	19.9	6.8	6.2	7.5	15.0	7.3	8.1	**0.00**

### ﻿Statistical analyses

We compared the Lung Cu population to its closest relatives *Hemiphyllodactylushuishuiensis* and *H.yanshanensis* from southern China and to other species in clade 6. *Hemiphyllodactylusdushanensis* was not evaluated due to the absence of comparative morphological data. Morphological data used for the statistical analyses were obtained from the Lung Cu population (seven specimens) and data from 76 specimens of 12 other species available from previous studies (Ngo et al. 2014; Nguyen et al. 2014; Yan et al. 2016; Sung et al. 2018; Eliades et al. 2019; Nguyen et al. 2020; Do et al. 2020; Zhang et al. 2020; Agung et al. 2022). Raw data for *H.typus* (four specimens [cat. Nos. CUMZ. R 2013.19; RBWS 19309; JAV bg. R.39; MS92002]) was obtained from the La Sierra University Herpetological Collection, La Sierra University, Riverside, California, USA. Museum Abbreviations: CUMZ - Museum of Zoology, Chulalongkorn University, Bangkok, Thailan; JAV-J.-M. VINSON; MS-Montri Sumontha; RBWS: Royal Belgian Institute of Natural Sciences. The raw morphological data are provided in Tables 3, 4.

**Table 3.** Morphometric and categorical data used in the analyses from specimens of *Hemiphyllodactylus* members within clade 6; m = male; f = female.

Species	Museum no.	Sex	Morphometric data								Categorical data			
			SVL	TrunkL	HeadL	HeadW	SnEye	NarEye	EyeD	SnW	PosOStrp	BodPartn	PosSacrlMakg	PosOTrSpt
*banaensis*	ITBCZ 2465	m	47.3	21.7	11.2	7.2	4.2	3.2	2.7	1.5	No	Yes	Yes	No
	ITBCZ 2461	m	48.2	23.8	10.8	7.6	4.5	3.5	2.7	1.5	No	Yes	Yes	No
	ITBCZ 2450	f	48.3	23.8	10.7	7.2	4	3.3	2.7	1.6	No	Yes	Yes	No
	ITBCZ 2462	f	50.9	24.4	12	7.9	4.6	3.6	2.7	1.7	No	Yes	Yes	No
	ITBCZ 2463	f	50	23.7	11.3	7.4	4.1	3.5	2.5	1.7	No	Yes	Yes	No
	ITBCZ 2464	f	51	24.1	12	7.9	4.5	3.6	2.6	1.8	No	Yes	Yes	No
	ITBCZ 2466	f	50.6	24.4	11.5	7.9	5.2	3.5	2.5	1.7	No	Yes	Yes	No
	ITBCZ 2467	f	50.1	22.3	11	7.5	4.4	3.1	2.4	1.7	No	Yes	Yes	No
	ITBCZ 2468	f	45.2	20.4	10	7	3.8	2.8	2.1	1.5	No	Yes	Yes	No
	ITBCZ 2469	f	49	23	10.9	7.4	4.3	3.4	2.7	1.8	No	Yes	Yes	No
*bonkowskii*	IEBR 4689	m	45.8	23.6	10.5	8.6	4.9	3.2	3.1	1.9	Yes	Yes	Yes	Yes
	IEBR 4690	m	40.7	20.9	9.6	7.9	4	3.1	2.9	1.9	Yes	Yes	Yes	Yes
	IEBR 4691	f	44.9	21.5	10.3	8.5	4.7	3.3	2.7	2.1	Yes	Yes	Yes	Yes
	IEBR 4693	f	45.4	22.5	10.5	9.2	5.1	3.6	3.1	2.1	Yes	Yes	Yes	Yes
	IEBR 4692	f	48	26.7	11.6	9.4	5.2	3.6	3.1	2.4	Yes	Yes	Yes	Yes
	IEBR 4749	f	36.6	17.6	8.9	7.1	4	2.9	2.3	1.7	Yes	Yes	Yes	Yes
*dupanglingensis*	CSUFT 00401	f	40.8	18.7	9.8	7.5	4.5	3.3	2.5	1.8	Yes	Yes	No	Yes
	CSUFT 00402	m	41	19.4	11.2	7.5	4.7	3.2	2.6	1.5	Yes	Yes	No	Yes
	CSUFT 00403	m	39.5	17.2	10.3	7.2	4.2	3	2.5	1.4	Yes	Yes	No	Yes
	CSUFT 00404	m	40.8	19.7	11.1	7.6	4.3	3.1	2.7	1.6	Yes	Yes	No	Yes
	CSUFT 00405	m	39.4	18.1	10.5	6.8	4.3	3.2	2.5	1.7	Yes	Yes	No	Yes
	CSUFT 00406	m	44.7	22.8	10.9	7.8	4.6	3.3	2.7	1.7	Yes	Yes	No	Yes
*hongkongensis*	SYS r001735	m	33.6	15.8	9.3	6.9	3.1	2.4	2	1.1	Yes	Yes	No	Yes
	SYS r001728	f	37.5	19.4	9.5	5.2	3.2	2.6	2.2	1.2	Yes	Yes	No	Yes
	SYS r001729	f	37.4	19.3	9.9	6.4	3.7	2.5	2.3	1.1	Yes	Yes	No	Yes
	SYS r001730	f	40.8	21	10.4	8	3.8	2.9	2.4	1.2	Yes	Yes	No	Yes
	SYS r001731	f	42.1	21.3	10.3	7.5	3.6	3.1	2.3	1.3	Yes	Yes	No	Yes
	SYS r001732	f	43	22	11.2	8.2	4	3	2.5	1.4	No	No	No	Yes
	SYS r001733	f	38.9	20.4	10.4	8.2	3.6	3	2.3	1.3	Yes	Yes	No	Yes
	SYS r001734	m	32.3	15.6	8.7	7	3.2	2.6	2.3	1.1	Yes	Yes	No	Yes
*huishuiensis*	NJNUh00851	m	42.6	20.5	9.5	7.8	3.9	2.8	2.7	1.2	Yes	Yes	Yes	No
	NJNUh00859	m	47.4	22.4	13.9	8.5	4.4	3.1	2.7	1.4	Yes	Yes	Yes	No
	NJNUh00852	f	44.3	22.3	9.8	7.5	3.8	2.8	2.5	1.2	Yes	Yes	Yes	No
	NJNUh00854	f	51.2	25	10.8	8.8	4.8	3.6	2.7	1.5	Yes	Yes	Yes	No
	NJNUh00855	f	49.3	24.9	11.9	8.4	4.8	3.2	2.8	1.2	Yes	Yes	Yes	No
	NJNUh00856	f	36.2	17.3	8.4	7.1	3.1	2.5	2.4	1	Yes	Yes	Yes	No
	NJNUh00857	f	45.5	21.5	10.1	8	4.2	3.4	2.4	1.1	Yes	Yes	Yes	No
	NJNUh00858	f	35.7	17.4	8.4	6.3	3.7	2.5	2.3	1.1	Yes	Yes	Yes	No
*kiziriani*	IEBR A.2014.3	m	40.1	19.7	7.4	6.7	4.2	3.2	2.7	1.7	Yes	Yes	Yes	Yes
	IEBR A.2014.4	m	35.1	16.4	6	6.3	3.8	3	2.5	1.3	Yes	Yes	Yes	Yes
	VNMN A.2014.1	m	37.6	18.8	6.7	6.9	3.5	2.7	2.4	1.4	Yes	Yes	Yes	Yes
	ZFMK 95702	m	36.2	18.8	6.3	7.2	3.8	2.8	2.7	1.3	Yes	Yes	Yes	Yes
*kiziriani*	IEBR A.2014.5	f	40.8	20.7	7.1	6.8	3.9	3.1	2.85	1.4	Yes	Yes	Yes	Yes
	NUOL R-2014.1	f	36.3	16.4	6.1	6.7	3.8	2.9	2.4	1.3	Yes	Yes	Yes	Yes
	NUOL R-2014.2	f	40	20.1	6.3	7.4	4	3	2.4	1.3	Yes	Yes	Yes	Yes
	VNMN A.2014.2	f	40.8	19.2	7.3	6.8	4.1	3.3	2.6	1.4	Yes	Yes	Yes	Yes
	ZFMK 95703	f	36.7	19	6.2	6.6	3.8	3	2.4	1.4	Yes	Yes	Yes	Yes
	ZFMK 95704	f	40.1	22	7.3	7.3	4.4	3.3	2.7	1.5	Yes	Yes	Yes	Yes
*Hemiphyllodactyluslungcuensis* sp. nov.	VNUF R.2021.01	m	39.2	20.3	11	8	4.3	3.2	1.8	1.5	Yes	Yes	Yes	Yes
	IEBR R.5151	m	43	23	11.6	8	4.5	2.9	2.1	2	Yes	Yes	Yes	Yes
	VNUF R.2021.03	m	44.2	23.1	11.1	7	5	3.6	2.4	1.7	Yes	Yes	Yes	Yes
	IEBR R.5152	f	39.2	19	8.5	7.3	4.1	3.2	2.5	1.9	Yes	Yes	Yes	Yes
	VNUF R.2021.08	f	43.5	22.3	10.3	8	4.2	3.3	2.1	2.1	Yes	Yes	Yes	Yes
	VNUF R.2023.01	m	35.3	17	8.9	6.9	3.6	3.13	1.8	1.4	Yes	Yes	Yes	Yes
	VNUF R.2023.02	f	37.4	19.3	8.5	7	3.9	3	1.8	1.4	Yes	Yes	Yes	Yes
*nahangensis*	IEBR 4741	m	43.6	21.3	10.7	7.4	3.8	3.1	2.5	1.3	No	Yes	Yes	Yes
	IEBR 4742	m	43.5	21.3	10.2	7.1	4	3.2	2.6	1.1	No	Yes	Yes	Yes
	IEBR 4743	f	41.4	20.9	10	6.9	3.5	2.8	2.7	1	No	Yes	Yes	Yes
*ngocsonensis*	IEBR 4694	m	45.5	23.6	9.8	8.5	4.5	3.3	2.8	1.7	Yes	No	Yes	Yes
	IEBR 4695	m	45.9	23.7	10.2	8.2	4.9	3.8	2.8	1.8	Yes	No	Yes	Yes
	IEBR 4696	f	46	23.1	10.5	8.6	4.6	3.3	2.7	1.7	Yes	No	Yes	Yes
	IEBR 4697	f	46.9	22.6	10.5	8.5	4.6	3.1	2.8	1.8	Yes	No	Yes	Yes
*serpispecus*	NUOL 00476	m	41.9	21.4	10.4	7.7	4	3.3	2.4	1.5	Yes	Yes	Yes	Yes
*typus*	JAV bg. R.39	f	34.3	18	8	5.3	3.3	2.5	1.7	1.2	Yes	Yes	Yes	Yes
	MS92002	f	34.5	20.1	9.1	5	3.5	2.6	2.1	0.7	Yes	Yes	Yes	Yes
	CUMZ R 2003.19	f	36.3	22.1	8.1	4.9	3.1	2.5	2	1.1	Yes	Yes	Yes	Yes
	RBWS 19309	f	22.6	12.4	6.6	4.5	2.4	1.9	1.5	0.8	Yes	Yes	Yes	Yes
*yanshanensis*	KIZ 062090	m	40.0	18.9	7.3	7.7	4.1	3.2	2.3	1.5	Yes	Yes	Yes	No
	KIZ 062091	f	43.9	20.1	8.2	8.4	4.2	3.2	2.7	1.5	Yes	Yes	Yes	No
	KIZ 062092	f	46.3	22.3	8.3	8.6	4.6	3.5	2.4	1.6	Yes	Yes	Yes	No
	KIZ 062093	f	38.8	18.8	8.1	8.4	4.3	3.4	2.7	1.6	Yes	Yes	Yes	No
	KIZ 062094	f	40.8	19.8	7.3	7.7	4.1	3.1	2.3	1.4	Yes	Yes	Yes	No
	KIZ 062095	f	41.2	20.1	7.5	8.1	4.5	3.1	2.2	1.6	Yes	Yes	Yes	No
	KIZ 062096	f	42.6	19.3	7.8	8.3	4.2	3.2	2.6	1.5	Yes	Yes	Yes	No
	KIZ 062097	m	43.5	19.7	7.4	8.6	4.1	3.1	2.3	1.5	Yes	Yes	Yes	No
	KIZ 062098	m	38.6	18.2	7.3	7.7	3.8	3.0	2.5	1.5	Yes	Yes	Yes	No
	KIZ 062099	m	34.0	17.4	6.	6.4	3.5	2.4	1.7	1.3	Yes	Yes	Yes	No
	KIZ 062100	f	37.0	17.7	6.8	7.6	3.7	3.0	2.4	1.4	Yes	Yes	Yes	No
	KIZ 062101	f	43.1	19.5	8.1	8.2	4.3	3.4	2.6	1.7	Yes	Yes	Yes	No
	KIZ 062102	f	41.1	19.9	7.5	7.8	4.3	3.4	2.3	1.5	Yes	Yes	Yes	No
*zugi*	IEBR A.2013.20	m	44.6	22.4	10.1	7.5	4.9	3.1	2.9	1.7	Yes	Yes	No	Yes
	ZFMK 94781	m	38.3	21.4	9.2	6.9	4.1	2.9	2.6	1.5	Yes	Yes	No	Yes
	IEBR A.2013.21	m	40.1	20.8	9	7.1	4.1	2.6	2.7	1.5	Yes	Yes	No	Yes

**Table 4.** Meristic data used in the analyses from specimens of *Hemiphyllodactylus* members within clade 6; m = male; f = female.

Species	Museum no.	Sex	Meristic data							
			Chin	CN	SL	IL	VS	DS	SL1F	SL1T
*banaensis*	ITBCZ 2465	m	6	3	11	10	9	18	5	5
	ITBCZ 2461	m	6	3	11	9	10	18	5	5
	ITBCZ 2450	f	6	3	11	10	10	18	5	5
	ITBCZ 2462	f	7	3	12	11	11	20	5	5
	ITBCZ 2463	f	6	3	9	10	10	18	5	5
	ITBCZ 2464	f	6	3	10	10	10	17	5	5
	ITBCZ 2466	f	6	3	10	10	9	17	5	5
	ITBCZ 2467	f	6	3	12	11	10	19	5	4
	ITBCZ 2468	f	6	3	11	11	12	20	5	5
	ITBCZ 2469	f	7	3	11	11	10	20	5	5
*bonkowskii*	IEBR 4689	m	7	3	10	10	13	24.5	5	5
	IEBR 4690	m	7	3	9	9	15	25	5	5
	IEBR 4691	f	5	3	9	9	15	25	5	5
	IEBR 4693	f	7	3	9.5	8	15	25.5	5	4
	IEBR 4692	f	7	3	10	10.5	14	25	5	5
	IEBR 4749	f	6	3	10	9	14	26	5	5
*dupanglingensis*	CSUFT 00401	f	10	4	11	12	11	15	5	5
	CSUFT 00402	m	10	3	11	9	11	15	4	4
	CSUFT 00403	m	9	4	11	9	10	16	5	4
	CSUFT 00404	m	11	3	12	10	10	15	5	5
	CSUFT 00405	m	11	3	11	10	11	14	5	5
	CSUFT 00406	m	11	3	14	9	11	14	5	5
*hongkongensis*	SYS r001735	m	5	3	10	9.5	10	15	5	5
	SYS r001728	f	6	3	11	10	10	13	4	5
	SYS r001729	f	5	3	12	10	9	14	4	5
	SYS r001730	f	6	3	12	10	9	13	4	5
	SYS r001731	f	6	3	10	10	9	12	3	5
	SYS r001732	f	5	4	11.5	10.5	9	14	4	5
	SYS r001733	f	6	3	11	10.5	10	13	4	5
	SYS r001734	m	6	3	11	10.5	9	14	5	5
*huishuiensis*	NJNUh00851	m	10	3	11	11	9	14	3	3
	NJNUh00859	m	8	3	11	10.5	9	15	3	3
	NJNUh00852	f	10	3	10	10	8	13	3	3
	NJNUh00854	f	10	3	9	9	7	13	3	3
	NJNUh00855	f	10	3	10	9	8	13	3	3
	NJNUh00856	f	10	3	10	10	7	13	3	3
	NJNUh00857	f	8	3	10	10	8	14	3	3
	NJNUh00858	f	10	3	10	10	9	14	3	3
*kiziriani*	IEBR A.2014.3	m	9	4	11	10.5	13.5	21.5	5	5
	IEBR A.2014.4	m	9	4	11	9	14.5	26	5	5
	VNMN A.2014.1	m	7	4	10	10	13.5	22.5	5	5
	ZFMK 95702	m	6	4	10.5	9	14	23.5	5	5
	IEBR A.2014.5	f	6	4	10.5	10	11.5	20.5	5	5
*kiziriani*	NUOL R-2014.1	f	9	4	11	10.5	13.5	23.5	5	5
	NUOL R-2014.2	f	6	4	10	10	11	18	5	5
	VNMN A.2014.2	f	8	4	11	9	12	24	5	5
	ZFMK 95703	f	8	4	10	10.5	13	25	5	5
	ZFMK 95704	f	9	4	10.5	9.5	12.5	23.5	5	5
*Hemiphyllodactyluslungcuensis* sp. nov.	VNUF R.2021.01	m	8	3	12	10.5	7	13	4	4
	IEBR R.5151	m	8	3	11	11	6	13	4	4
	VNUF R.2021.03	m	9	3	12	10	7	15	4	4
	IEBR R.5152	f	9	2	11	10	9	16	4	4
	VNUF R.2021.08	f	10	3	11	10	7	12	3	4
	VNUF R.2023.01	m	10	3	11.5	9.5	10	17	4	4
	VNUF R.2023.02	f	10	3	11	9.5	11	16	4	4
*nahangensis*	IEBR 4741	m	8	3	11	9.5	9	22	3	3
	IEBR 4742	m	8	2.5	11.5	10.5	9	18	3	3
	IEBR 4743	f	9	3	11	10.5	13	23	3	3
*ngocsonensis*	IEBR 4694	m	8	3	10.5	10	14	20.5	5	5
	IEBR 4695	m	6	3	10	10	13	20.5	4	5
	IEBR 4696	f	8	3	10	9	15	19.5	5	5
	IEBR 4697	f	8	3	10	9.5	14	19.5	5	6
*serpispecus*	NUOL 00476	m	7	3	11	9	10	26	4	4
*typus*	JAV bg. R.39	f	10	6	10.5	10	9	14	4	4
	MS92002	f	15	5	12	11	12	15	4	4
	CUMZ R 2003.19	f	11	5	11.5	11	11	15	4	4
	RBWS 19309	f	15	5	11.5	11	12	16	4	5
*yanshanensis*	KIZ 062090	m	9	6	9.5	10	8	14	5	5
	KIZ 062091	f	8	5	11.5	11	7	15	5	5
	KIZ 062092	f	11	5	11	10.5	8	15	5	5
	KIZ 062093	f	8	5	10.5	10	13	22	5	5
	KIZ 062094	f	9	5	12	9.5	7	14	5	5
	KIZ 062095	f	8	5	11	10.5	7	13	5	6
	KIZ 062096	f	8	5	11.5	11	8	15	4	5
	KIZ 062097	m	8	5	10.5	10	7	14	4	5
	KIZ 062098	m	9	5	10	9.5	8	18	5	5
	KIZ 062099	m	8	5	11	11	8	15	5	5
	KIZ 062100	f	8	5	10	9.5	11	20	5	5
	KIZ 062101	f	10	5	11	10	8	14	4	5
	KIZ 062102	f	10	5	10.5	9.5	7	11	4	5
*zugi*	IEBR A.2013.20	m	9	3	12.5	11	15	21.5	4	4.5
	ZFMK 94781	m	12	2	11	10.5	15	20.5	5	5
	IEBR A.2013.21	m	9	2.5	10.5	11	16	21.5	5	5

All morphological analyses were conducted in R v.4.2.1 (R Core Team, 2021). An ANOVA was conducted on morphometric and meristric characters with statistically similar variances (i.e. p values > 0.05 in a Levene’s test) to search for the presence of statistically significant mean differences (p < 0.05) across the data set. Characters bearing statistically significant differences were subjected to a TukeyHSD test to ascertain which population pairs differed significantly from each other for those particular characters. Boxplots were generated in order to visualize the range, mean, and degree of differences among species bearing statistically different mean values for sets of characters.

Morphometric characters used in the statistical analyses were SVL, TrunkL, HeadL, HeadW, SnEye, NarEye, EyeD, and SnW. Tail metrics were not used due to the high degree incomplete sampling (i.e., regenerated, broken, or missing). To remove potential effects of allometry on morphometric traits (sec. Chan and Grismer 2022), we used the following equation: Xadj= log(X)-β[log(SVL)-log(SVLmean)], where Xadj = adjusted value; X = measured value; β = unstandardized regression coefficient for each population; and SVLmean = overall average SVL of all populations (Thorpe 1975, 1983; Turan 1999; Lleonart et al. 2000, accessible in the R package GroupStruct (available at https://github.com/chankinonn/GroupStruct). The morphometrics of each species were normalized separately and then concatenated into a single data set so as not to conflate potential intra- with interspecific variation (Reist 1986; McCoy et al. 2006) (Table 3). Meristic characters (scale counts) used in statistical analyses were Chin, CN, SL, IL, VS, DS, SL1F, and SL1T (Table 4). Precloacal and femoral pores were excluded from the multivariate analyses due to their absence in females. Categorical characters analyzed were the presence or absence of BodPartn; PosOStrp; PosOTrSpt; and PosSacrlMakg (Table 3).

Morphospatial relationships based on the normalized morphometrics and meristics were visualized using principal component analysis (PCA) from the ADEGENET package in R (Jombart et al. 2010) to determine if their positioning was consistent with the putative species boundaries delimited by the molecular phylogenetic analyses and defined by the univariate analyses (see above). PCA, implemented using the “prcomp()” command in R, is an indiscriminate analysis plotting the overall variation among individuals (i.e. data points) while treating each individual independently (i.e. not coercing data points into pre-defined groups). Subsequent to the PCA, a discriminant analysis of principle components (DAPC) was used to test for corroboration and further discrimination of morphospatial differences among the putative species. DAPC*a priori* groups the individuals of each predefined population inferred from the phylogeny into separate clusters (i.e. plots of points) bearing the smallest within-group variance that produce linear combinations of centroids having the greatest between-group variance (i.e. linear distance; Jombart et al. 2010). DAPC relies on standardized data from its own PCA as a prior step to ensure that variables analyzed are not correlated and number fewer than the sample size. Principal components with eigenvalues accounting for 90–95% of the variation in the data set were retained for the DAPC analysis according to the criterion of Jombart et al. (2010).

To test and further corroborate the PCA and DAPC analyses, we conducted a multiple factor analysis (MFA) on the above mentioned morphological characters plus the categorical color pattern charactrers for a near total evidence data set (see Tables 5, 6). The MFA was implemented using the *mfa* () command in the R package *FactorMineR* (Husson et al. 2017) and visualized using the *Factoextra* package (Kassambara and Mundt 2017). MFA is a global, unsupervised, multivariate analysis that incorporates qualitative and quantitative data (Pagès 2015), making it possible to analyze different data types simultaneously in a nearly total evidence environment. In an MFA, each individual is described by a different set of variables (i.e. characters) which are structured into different data groups in a global data frame–in this case, quantitative data (i.e. meristics and normalized morphometrics) and categorical data (i.e. color pattern). In the first phase of the analysis, separate multivariate analyses are carried out for each set of variables–principal component analyses (PCA) for the quantitative data sets and a multiple correspondence analysis (MCA) for categorical data. The data sets are then normalized separately by dividing all their elements by the square root of their first eigenvalues. For the second phase of the analysis, the normalized data sets are concatenated into a single matrix for a global PCA. Standardizing the data in this manner prevents one data type from overleveraging another. In other words, the normalization of the data in the first phase prevents data types with the highest number of characters or the greatest amount of variation from outweighing other data types in the second phase. This way, the contribution of each data type to the overall variation in the data set is scaled to define the morphospatial distance between individuals as well as calculating the contribution of each data type and character to the overall variation in the data set (Pagès 2015; Kassambara and Mundt 2017).

**Table 5.** Significant p-values from the results of the ANOVA and Turkey HSD analyses comparing all combinations of species pairs. Abbreviations are in the Materials and methods.

Morphological characters	SVL	TrunkL	HeadL	HeadW	SnEye	NarEye	EyeD	SnW	Chin	CN	SL	VS	DS	SL1F	SL1T
*lungcuensis* sp. nov. vs. *banaensis*	0.002	<0.001	0.005				<0.001		<0.001				0.002	0.002	0.002
*typus* vs. *lungcuensis* sp. nov.	0.003	0.026		0.00	0.00	0.00		0.00		0.00					
*lungcuensis* sp. nov. vs. *kiziriani*		0.004	0.00				<0.001	<0.001		<0.001		<0.001	0.00		0.00
*ngocsonensis* vs. *lungcuensis* sp. nov.		<0.001		0.032			<0.001					<0.001	<0.001		<0.001
*typus-lungcuensis*		<0.001	<0.001						<0.001			0.026		0.003	
*yanshanensis* vs. *lungcuensis* sp. nov.		0.03					0.002			0.00					
*lungcuensis* sp. nov. vs. *bonkowskii*				0.008	<0.001		<0.001	0.02	0.002		0.002	0.00	0.00		0.00
*lungcuensis* sp. nov. vs. *hongkongensis*						<0.001		<0.001	<0.001						
*lungcuensis* sp. nov. vs. *huishuiensis*							<0.001	<0.001							
*nahangensis* vs. *lungcuensis* sp. nov.							<0.001	<0.001					<0.001		<0.001
*zugi* c *lungcuensis* sp. nov.							<0.001					<0.001	<0.001		<0.001
*lungcuensis* sp. nov. vs. *dupanglingensis*												0.027			
*serpispecus* vs. *lungcuensis* sp. nov.													<0.001		<0.001

**Table 6.** Summary statistics of morphometric and meristic characters among the *Hymiphyllodactylus* species in clade 6.

Species	SVL	TrunkL	HeadL	HeadW	SnEye	NarEye	EyeD	SnW	Chin	CN	SL	IL	VS	DS	SL1F	SL1T
***banaensis* (*n* = 10)**																
Mean	1.69	1.36	1.05	0.87	0.64	0.52	0.41	0.22	6.2	3	10.8	10.3	10.1	18.5	5	4.9
SD	0.017	0.014	0.012	0.009	0.027	0.021	0.031	0.019	0.422	0	0.919	0.674	0.876	1.179	0	0.316
Lower	1.66	1.34	1.03	0.86	0.60	0.48	0.36	0.19	6	3	9	9	9	17	5	4
Upper	1.71	1.39	1.07	0.89	0.69	0.56	0.45	0.26	7	3	12	11	12	20	5	5
***bonkowskii* (*n* = 6)**																
Mean	1.64	1.34	1.01	0.93	0.67	0.52	0.45	0.31	6.5	3	9.58	9.25	14.33	25.17	5	4.83
SD	0.043	0.020	0.011	0.012	0.019	0.020	0.027	0.023	0.837	0	0.491	0.880	0.816	0.516	0	0.408
Lower	1.56	1.32	1.00	0.91	0.63	0.48	0.42	0.27	5	3	9	8	13	24.5	5	4
Upper	1.68	1.37	1.03	0.95	0.69	0.54	0.49	0.33	7	3	10	10.5	15	26	5	5
***dupanglingensis* (*n* = 6)**																
Mean	1.61	1.28	1.03	0.87	0.65	0.50	0.41	0.21	10.33	3.33	11.67	9.83	10.67	14.83	4.83	4.67
SD	0.020	0.011	0.021	0.013	0.015	0.013	0.011	0.038	0.82	0.516	1.211	1.169	0.516	0.753	0.408	0.516
Lower	1.60	1.27	0.99	0.85	0.63	0.49	0.40	0.16	9	3	11	9	10	14	4	4
Upper	1.65	1.30	1.05	0.88	0.67	0.52	0.43	0.26	11	4	14	12	11	16	5	5
***hongkongensis* (*n* = 8)**																
Mean	1.58	1.29	1.00	0.85	0.55	0.44	0.36	0.08	5.63	3.13	11.06	10.13	9.38	13.5	4.13	5
SD	0.044	0.012	0.012	0.061	0.022	0.024	0.021	0.022	0.518	0.35	0.776	0.353	0.518	0.926	0.641	0
Lower	1.52	1.27	0.98	0.72	0.51	0.41	0.32	0.05	5	3	10	9.5	9	12	3	5
Upper	1.63	1.30	1.01	0.91	0.58	0.47	0.39	0.12	6	4	12	10.5	10	15	5	5
***huishuiensis* (*n* = 8)**																
Mean	1.64	1.33	1.01	0.89	0.61	0.47	0.41	0.08	9.5	3	10.13	9.94	8.13	13.63	3	3
SD	0.058	0.011	0.044	0.016	0.027	0.024	0.020	0.036	0.93	0	0.641	0.678	0.835	0.744	0	0
Lower	1.55	1.32	0.97	0.87	0.58	0.44	0.37	0.03	8	3	9	9	7	13	3	3
Upper	1.71	1.35	1.11	0.92	0.66	0.52	0.44	0.13	10	3	11	11	9	15	3	3
***kiziriani* (*n* = 10)**																
Mean	1.58	1.28	0.82	0.84	0.59	0.48	0.41	0.15	7.7	4	10.55	9.8	12.9	22.8	5	5
SD	0.025	0.024	0.019	0.019	0.021	0.021	0.024	0.031	1.337	0	0.438	0.632	1.125	2.312	0	0
Lower	1.55	1.25	0.78	0.81	0.55	0.44	0.37	0.100	6	4	10	9	11	18	5	5
Upper	1.61	1.32	0.85	0.87	0.63	0.51	0.45	0.22	9	4	11	10.5	14.5	26	5	5
***lungcuensis* (*n* = 7)**																
Mean	1.60	1.31	0.10	0.87	0.63	0.50	0.31	0.23	9.14	2.86	11.36	10.07	8.14	14.57	3.86	4
SD	0.037	0.013	0.039	0.026	0.022	0.028	0.049	0.046	0.900	0.378	0.476	0.535	1.864	1.902	0.378	0
Lower	1.55	1.29	0.94	0.83	0.59	0.45	0.27	0.16	8	2	11	9.5	6	12	3	4
Upper	1.65	1.33	1.06	0.91	0.66	0.54	0.41	0.30	10	3	12	11	11	17	4	4
***nahangensis* (*n* = 3)**																
Mean	1.63	1.33	1.01	0.85	0.58	0.48	0.42	0.05	8.33	2.83	11.17	10.17	10.33	21	3	3
SD	0.012	4.15E-05	0.008	0.007	0.013	0.007	0.006	0.034	0.577	0.289	0.289	0.577	2.309	2.646	0	0
Lower	1.62	1.33	1.00	0.84	0.57	0.47	0.41	0.017	8	2.5	11	9.5	9	18	3	3
Upper	1.64	1.33	1.02	0.86	0.59	0.49	0.42	0.09	9	3	11.5	10.5	13	23	3	3
***ngocsonensis* (*n* = 4)**																
Mean	1.66	1.37	1.01	0.93	0.66	0.53	0.44	0.24	7.5	3	10.13	9.63	14	20	4.75	5.25
SD	0.006	0.004	0.008	0.010	0.016	0.029	0.010	0.007	1	0	0.25	0.479	0.816	0.577	0.5	0.5
Lower	1.66	1.36	1.00	0.91	0.65	0.50	0.43	0.23	6	3	10	9	13	19.5	4	5
Upper	1.67	1.37	1.02	0.94	0.69	0.57	0.45	0.25	8	3	10.5	10	15	20.5	5	6
***serpispecus* (*n* = 1)**																
Mean	1.62	1.33	1.01	0.88	0.59	0.51	0.38	0.17	7	3	11	9	10	26	4	4
SD	NA	NA	NA	NA	NA	NA	NA	NA	NA	NA	NA	NA	NA	NA	NA	NA
Lower	1.62	1.33	1.01	0.88	0.59	0.51	0.38	0.17	7	3	11	9	10	26	4	4
Upper	1.62	1.33	1.01	0.88	0.59	0.51	0.38	0.17	7	3	11	9	10	26	4	4
***typus* (*n* = 4)**																
Mean	1.50	1.26	0.90	0.69	0.49	0.38	0.26	-0.03	12.75	5.25	11.36	10.75	11	15	4	4.25
SD	0.096	0.026	0.028	0.016	0.027	0.015	0.033	0.115	2.630	0.5	0.629	0.5	1.414	0.816	0	0.5
Lower	1.35	1.22	0.88	0.68	0.46	0.36	0.21	-0.19	10	5	10.5	10	9	14	4	4
Upper	1.56	1.28	0.94	0.72	0.52	0.40	0.29	0.07	15	6	12	11	12	16	4	5
***yanshanensis* (*n* = 13)**																
Mean	1.61	1.29	0.87	0.90	0.62	0.50	0.38	0.18	8.77	5.077	10.77	10.15	8.23	15.38	4.69	5.08
SD	0.035	0.012	0.021	0.017	0.018	0.024	0.039	0.019	1.013	0.277	0.696	0.591	1.787	2.959	0.480	0.277
Lower	1.53	1.27	0.84	0.87	0.59	0.46	0.31	0.16	8	5	9.5	9.5	7	11	4	5
Upper	1.67	1.31	0.93	0.94	0.65	0.55	0.45	0.21	11	6	12	11	13	22	5	6
***zugi* (*n* = 3)**																
Mean	1.61	1.33	0.97	0.86	0.64	0.46	0.44	0.19	10	2.5	11.33	10.83	15.33	21.17	4.67	4.83
SD	0.034	0.010	0.012	0.001	0.013	0.031	0.001	0.009	1.732	0.5	1.041	0.289	0.577	0.577	0.577	0.289
Lower	1.58	1.32	0.96	0.85	0.62	0.42	0.436	0.185	9	2	10.5	10.5	15	20.5	4	4.5
Upper	1.65	1.34	0.98	0.86	0.65	0.48	0.438	0.202	12	3	12.5	11	16	21.5	5	5

## ﻿Results

### ﻿Phylogenetic analyses

**Figure 2.**
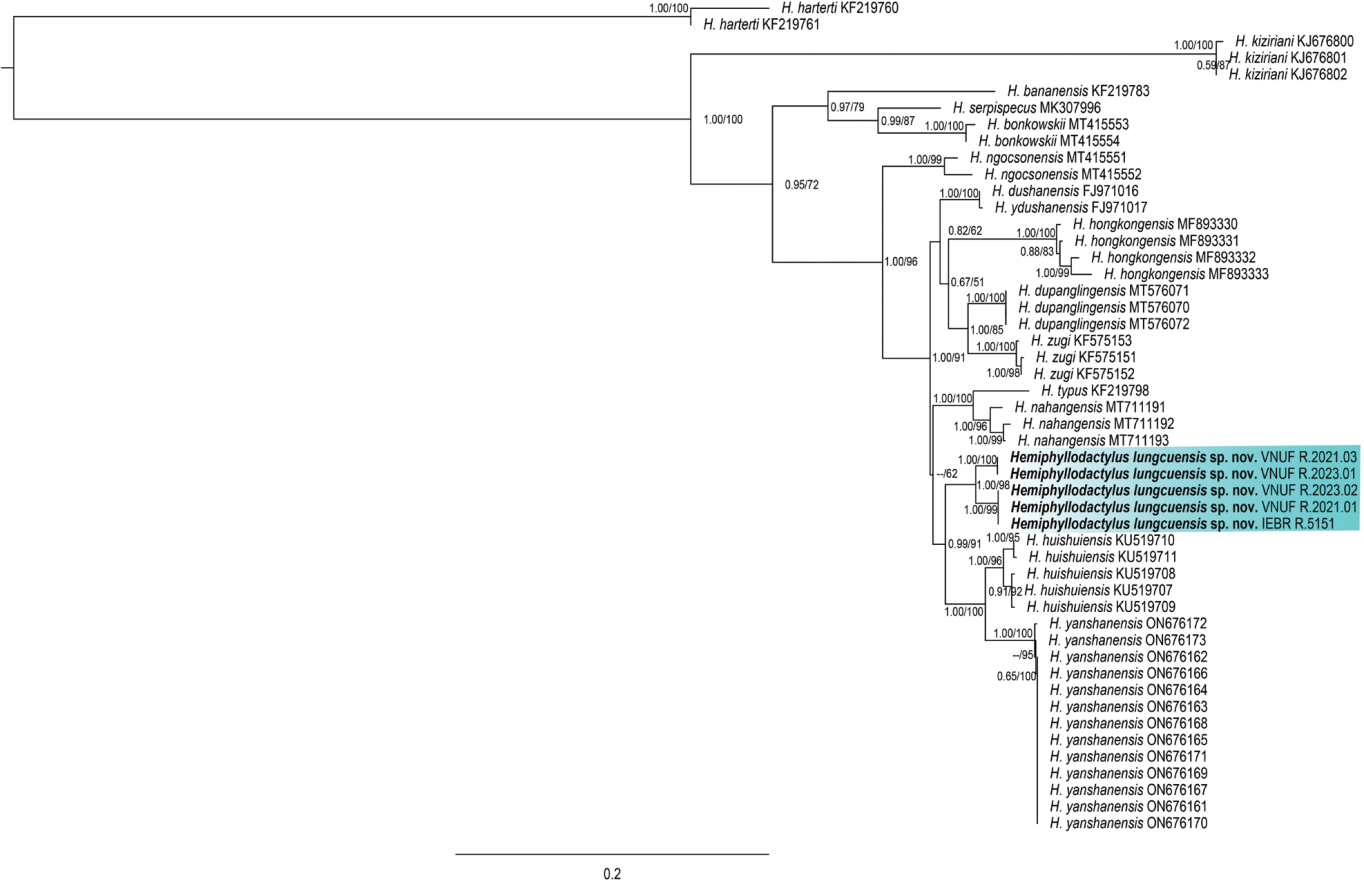
Maximum likelihood consensus tree of clade 6 (excepting *Hemiphyllodactylusharterti* used to root the tree following Grismer et al. 2013) of *Hemiphyllodactylus* with Bayesian posterior probabilities (BPP) and Ultrafast Bootstrap support (UFB) value coding at the nodes, respectively.

The ML and BI analyses returned well-supported, nearly identical topologies for all the known species of clade 6 (Agung et al. 2022) and were concordant with genus-wide phylogenies from previous analyses (Grismer et al. 2013; Grismer et al. 2020; Agung et al. 2022). The specimens from Lung Cu Commune, Dong Van District, Ha Giang Province, northeastern Vietnam, formed a monophyletic lineage in the *typus* group (Grismer et al. 2013) embedded within a larger lineage of Indochinese species where it is nested within clade 6. Herein, it was recovered as the well-supported (BI 0.99/ML 91) sister species to a lineage comprised of *H.huishuiensis* and *H.yanshanensis* (Fig. 2). The Lung Cu lineage differs from *H.huishuiensis* and *H.yanshanensis* by having uncorrected average pairwise sequence divergences of 5.3% and 6.8%, respectively (Table 2). In terms of genetic distance, the Lung Cu population is similar to *H.nahangensis* and *H.dushanensis*, but it has a genetic distance of 4.6% and 4.8% from *H.nahangensis* and *H.dushanensis*, respectively. Additionally, significant differences of morphometric and meristic traits among them are indicated in the comparisons. As such we hypothesize this population represents a new evolutionary lineage and is described as a new species below.

### ﻿Statistical analyses

**Figure 3.**
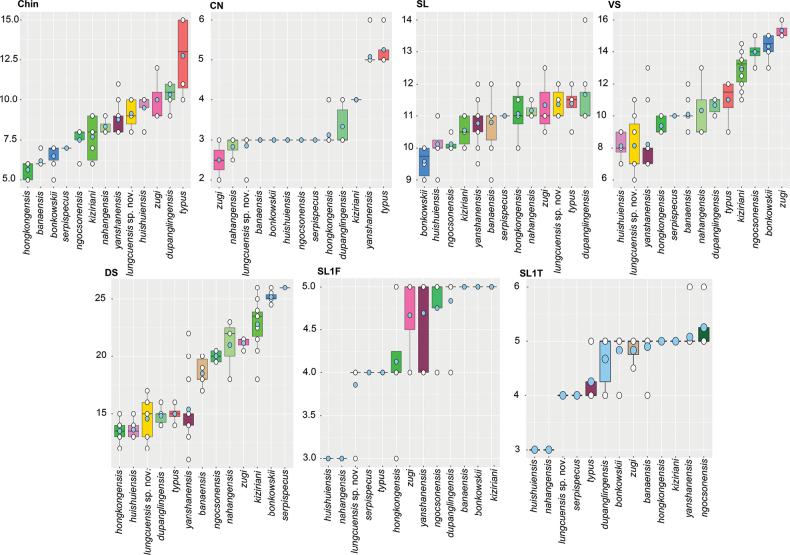
Boxplot comparisons of significantly different meristic characters between the Lung Cu population and other *Hemiphyllodactylus* species of clade 6. Pale blue circles are means and the black horizontal bars are medians.

**Figure 4.**
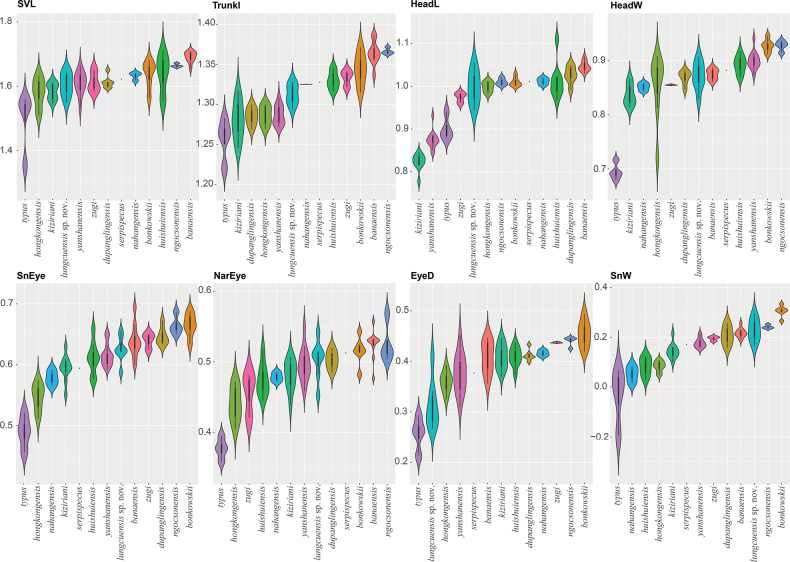
Violin plots of the significantly different morphometric characters between the Lung Cu population and other *Hemiphyllodactylus* species of clade 6 overlain with box plots showing the range, frequency, mean (white dot), and 50% quartile (black rectangle) of the size-adjusted morphometric and meristic characters. New species in bold.

The ANOVA and TukeyHSD *post hoc* tests of the adjusted morphometric and meristic characters were consistent with the phylogenetic results and their high degree of pairwise genetic distance among the species in recovering a number of statistically significant mean differences between the Lung Cu population and all other species (Tables 5, 6). Variation in all morphometric and metric characters are visualized in Figs 3, 4.

**Figure 5.**
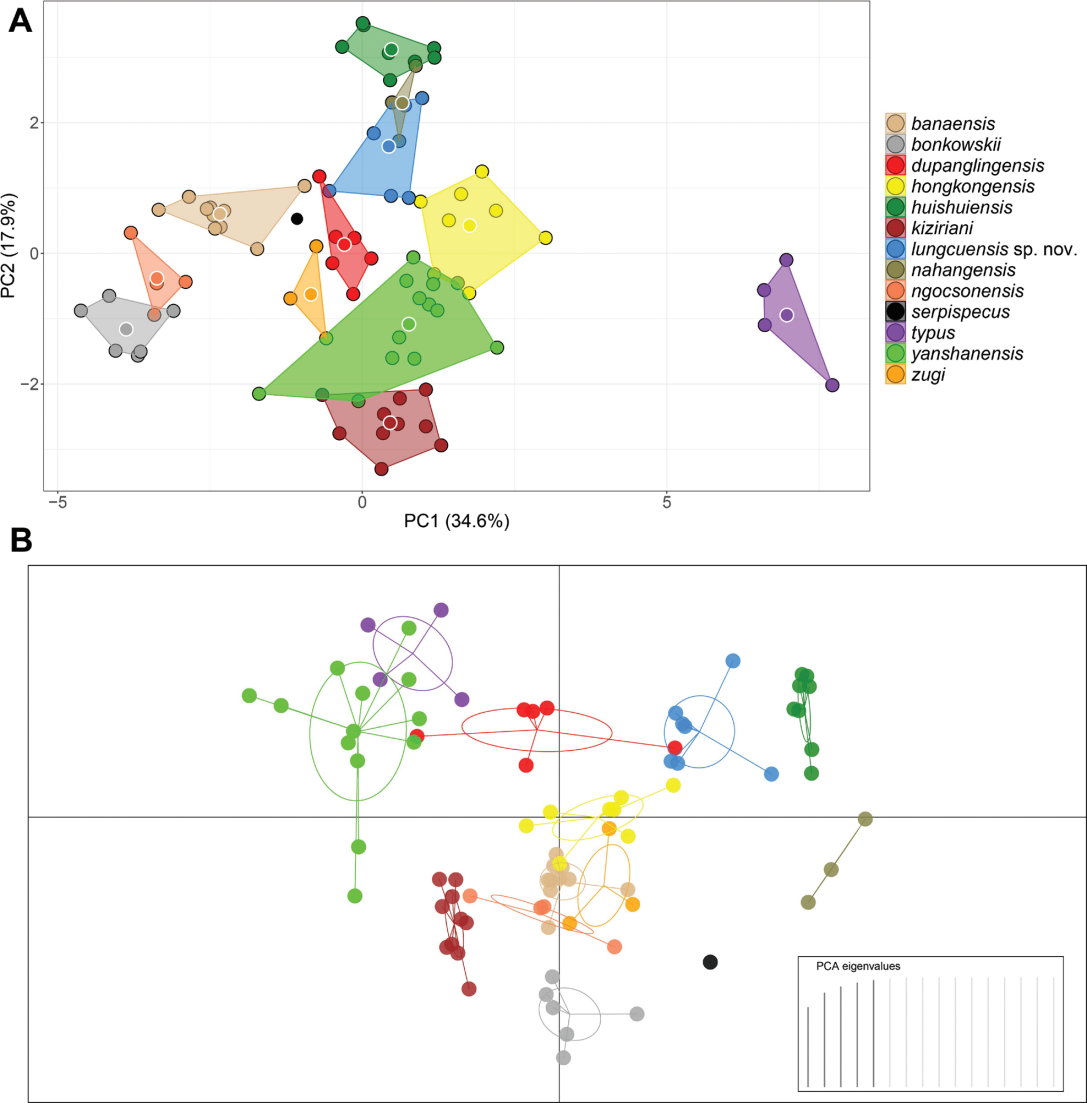
**A** principal component analysis (PCA) of *Hemiphyllodactylus* species of clade 6 showing their morphospatial relationships along the first two principal components **B** discriminant analysis of principal components (DAPC) based on retention of the first five PCs accounting for 98% of the variation.

The clustering of species in the PCA of clade 6 showed the first two principal components (PC1 and PC2) recovered 52.5% of the variation in the normalized morphometric and meristic data set (Fig. 5A). PC1 accounted for 34.6% of the variation in the data set and loaded most heavily for trunk length (TrunkL), head width (HeadW), snout-eye length (SnEye), nares-eye length (NarEye), and eye diameter (EyeD). PC2 accounted for an additional 17.9% of the data set and loaded most heavily for head length (HeadL), circumnasal scales (CN), dorsal scales (DS), the number of subdigital lamellae wider than long on the first finger (SL1F), and the number of subdigital lamellae wider than long on the first toe (SL1T) along PC2 (Fig. 5A, Table 7). The PCA recovered *Hemiphyllodactyluslungcuensis* sp. nov. to be widely separated from most other species and only overlapping with the distantly related *H.nahangensis* and minimally overlapping with *H.dupanglingensis*. The new species is well-separated from most other species in the DAPC and only slightly overlapping with the distantly related *H.dupanglingensis* (Fig. 5B). More importantly, its 66% confidence ellipse does not overlap with that of any other species.

**Table 7.** Summary statistics of the principal component analysis for *Hemiphyllodactylus* species.

	**PC1**	**PC2**	**PC3**	**PC4**	**PC5**	**PC6**	**PC7**	**PC8**
**Standard deviation**	2.354041057	1.693896742	1.274819788	1.167631895	1.035772699	0.858583005	0.792572769	0.700362564
**Proportion of Variance**	0.34634	0.17933	0.10157	0.08521	0.06705	0.04607	0.03926	0.03066
**Cumulative Proportion**	0.34634	0.52567	0.62725	0.71246	0.77951	0.82558	0.86484	0.8955
**eigen**	5.541509296	2.869286174	1.625165491	1.363364241	1.072825085	0.737164777	0.628171593	0.49050772
**SVL**	-0.275684939	0.230671227	-0.183268856	0.035839657	-0.118110503	0.272234498	-0.353548073	0.532231802
**TrunkL**	-0.308028087	0.226577926	0.113182	0.165672749	-0.09837754	0.21375662	0.23148674	0.352374067
**HeadL**	-0.182206073	0.401679247	0.111064477	0.286694098	-0.082466549	-0.270566918	0.230170931	-0.024756545
**HeadW**	-0.316234879	0.087646379	-0.266341502	-0.227593583	0.011593053	0.056636999	-0.034411085	-0.551718699
**SnEye**	-0.354053441	0.028071822	-0.174272185	0.028737797	0.370906143	-0.005790557	0.061744748	-0.038483117
**NarEye**	-0.343202425	0.013213311	-0.227705587	-0.097414199	0.248586294	-0.037810729	-0.050598906	0.110097139
**EyeD**	-0.316496337	-0.057054094	0.203121932	-0.054096772	0.11278894	0.198377934	-0.488753315	-0.270887117
**SnW**	-0.331682586	-0.118626	-0.220099451	0.078123766	0.10976895	-0.079025168	0.348298246	-0.089036833
**Chin**	0.209598984	0.041124515	0.055504502	0.015768954	0.762755038	0.126936754	0.252975725	0.142123111
**CN**	0.219334589	-0.306220475	-0.340275868	-0.238245722	0.106513528	0.269505437	-0.086774889	0.202680044
**SL**	0.150823615	0.072482948	-0.141526231	0.569582987	0.281130944	-0.34518498	-0.525400396	-0.03369833
**IL**	0.148019724	0.047098158	-0.160448641	0.539731081	-0.078204547	0.69478363	0.119615798	-0.305781505
**VS**	-0.159093977	-0.32427077	0.507208426	0.179912633	0.107284233	0.046759987	0.085474597	-0.042903745
**DS**	-0.216885816	-0.316665593	0.415917222	0.013670345	0.048885259	0.15087592	-0.139750976	0.074486639
**SL1F**	-0.160449559	-0.454450117	-0.164215408	0.230860029	-0.051722119	-0.131281578	0.073369609	0.169471363
**SL1T**	-0.088298003	-0.43959919	-0.276677158	0.243587056	-0.224574637	-0.154636155	0.081186695	0.024901385
	**PC9**	**PC10**	**PC11**	**PC12**	**PC13**	**PC14**	**PC15**	**PC16**
**Standard deviation**	0.645837646	0.528806731	0.4949505	0.460869402	0.422836018	0.392344947	0.348783481	0.252014728
**Proportion of Variance**	0.02607	0.01748	0.01531	0.01328	0.01117	0.00962	0.0076	0.00397
**Cumulative Proportion**	0.92157	0.93905	0.95436	0.96763	0.97881	0.98843	0.99603	1
**eigen**	0.417106265	0.279636559	0.244975998	0.212400606	0.178790298	0.153934558	0.121649916	0.063511423
**SVL**	-0.007653797	0.504865284	0.023054857	0.048923188	-0.104515394	-0.134955096	0.216496623	-0.075553468
**TrunkL**	-0.155806654	-0.402668791	0.169935171	-0.50108859	0.030208721	0.261221952	-0.105888367	0.183446071
**HeadL**	-0.196823028	-0.042928589	0.308909057	0.583413488	-0.235482569	0.083561907	-0.07755344	-0.18027682
**HeadW**	-0.057355934	0.018795152	0.420271	-0.230081521	-0.169918109	0.020732474	0.445117482	0.004175194
**SnEye**	0.006655124	-0.05473374	0.125911056	-0.081337071	0.26677457	-0.615512405	-0.423767342	-0.211307545
**NarEye**	0.209982569	-0.475190325	-0.461292899	0.279036274	-0.29657524	-0.040045604	0.211899966	0.218583377
**EyeD**	-0.467694393	0.020586761	-0.201246365	0.150990454	0.146331073	0.329187066	-0.274245212	0.024651913
**SnW**	0.373629734	0.470794413	-0.10280995	-0.013393282	0.031119697	0.456870887	-0.272519277	0.133686181
**Chin**	-0.293197569	0.139059923	-0.038969703	-0.051313747	-0.052718431	0.18026194	0.28186905	-0.211579231
**CN**	-0.036235154	-0.135400518	0.485735592	0.236559781	-0.276203918	0.132252018	-0.350233189	0.158104094
**SL**	0.189523962	-0.059900251	0.171907156	-0.215076887	-0.077003357	0.119789393	-0.02904125	0.125441842
**IL**	0.112573099	-0.066456621	-0.117163024	0.149019989	0.026731134	-0.082213823	0.038834193	-0.012764391
**VS**	-0.060117023	0.224584718	0.102501327	-0.019925796	-0.380657113	-0.333667822	0.032368952	0.484590421
**DS**	0.471295976	-0.169334813	0.207180516	0.049104744	-0.028195629	0.15739267	0.109826908	-0.543616511
**SL1F**	-0.192515088	-0.071305429	0.182854605	0.25220064	0.565596931	0.028793369	0.377898255	0.17530195
**SL1T**	-0.364000904	-0.005236656	-0.226318873	-0.228651157	-0.413553347	-0.023541078	-0.058595931	-0.412884904

**Figure 6.**
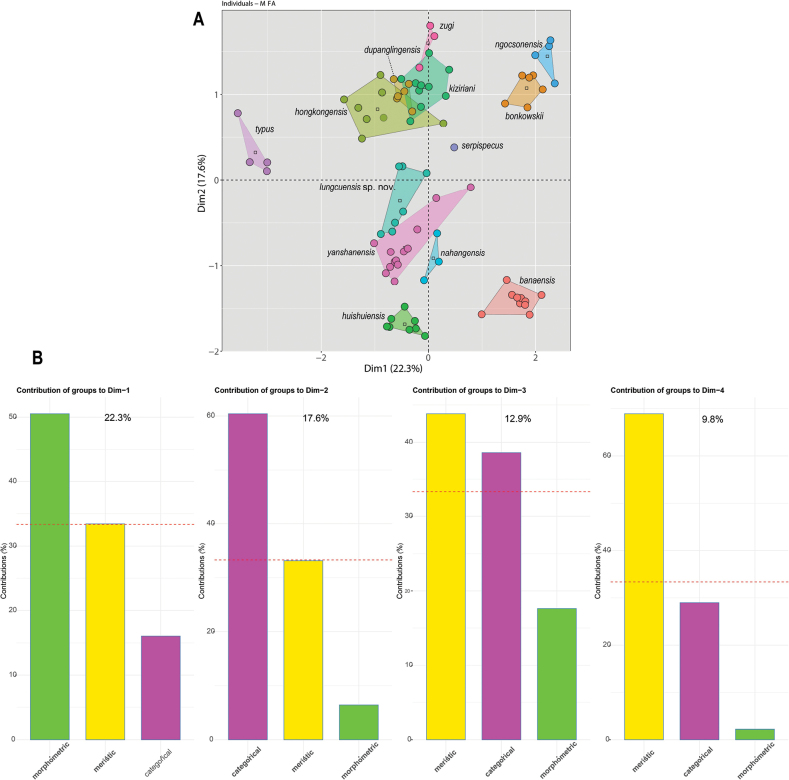
**A**MFA scatter plot showing the morphospatial relationships among the *Hemiphyllodactylus* species of clade 6 **B** bar graphs showing the percent contribution of each data type to the overall variation in the data set for the first four dimensions. The dashed red line in the bar graphs indicates the expected average value if the contributions of each data type were equal.

The MFA analysis recovered all species as being separated from one another including *Hemiphyllodactyluslungcuensis* sp. nov. and *H.nahangensis* (Fig. 6A). The normalized morphometric data contributed to approximately 50% of the variation along Dim-1 followed by the meristic and categorical data. For Dim-2, the categorical data contributed 60% of the variation followed by meristic and mensural data. Dim-3 showed that meristic data contributed 45% of the variation followed by categorical and mensural data. Finally, Dim-4 showed that meristic data contributed 70% of the variation followed by categorical and mensural data (Fig. 6B).

### ﻿Taxonomy

#### ﻿ Hemiphyllodactylus lungcuensis sp. nov.

Taxon classificationAnimaliaSquamataGekkonidae

64879394-8DB8-5685-9534-C5A76EAF82B1

https://zoobank.org/7E96A770-35D0-4B50-82B5-F8C7A4A817FD

**Figure 7.**
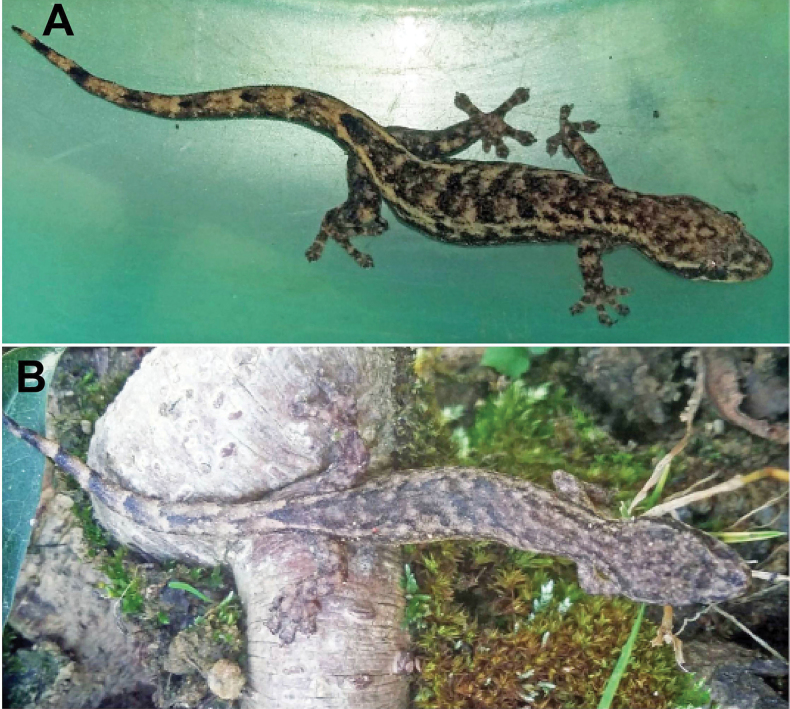
Dorsal views of *Hemiphyllodactyluslungcuensis* sp. nov. in life from Ha Giang Province, northeastern Vietnam **A** adult male holotype VNUF R.2021.01 **B** adult female paratype VNUF R.2021.08.

**Figure 8.**
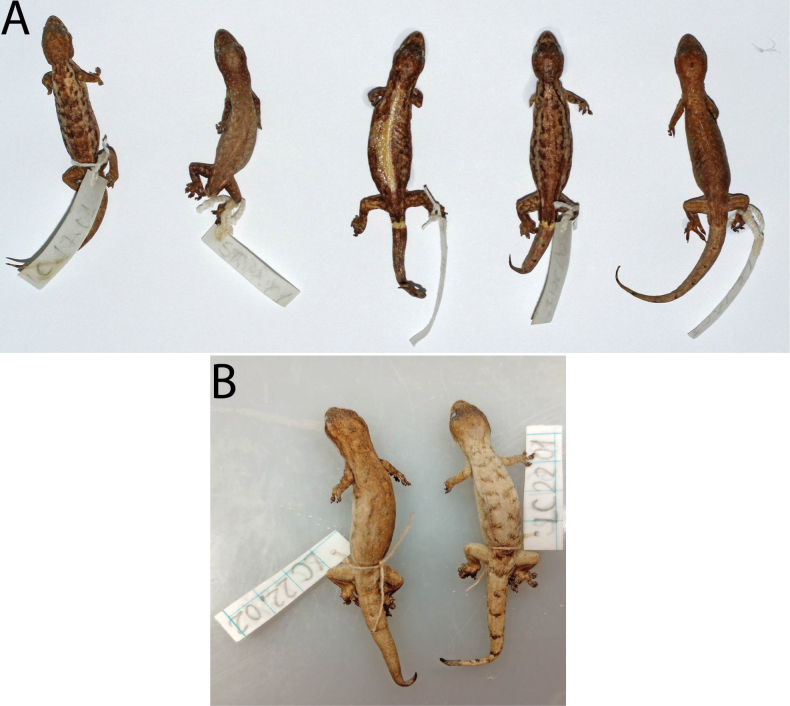
Type series of *Hemiphyllodactyluslungcuensis* sp. nov. in preservative from Ha Giang Province, northeastern Vietnam. A from right to left: Adult male holotype (VNUF R.2021.01), adult female paratype (IEBR R.5251), adult female paratype (VNUF R.2021.08), adult male paratype (IEBR R.5151), adult male paratype (VNUF R.2021.3). B From right to left: Adult female paratype (VNUF R.2023.02), and adult female paratype (VNUF R.2023.01).

Figs 7 , 8 Verbatim name. Lungcu Slender Gecko

##### Material examined.

***Holotype*.**VNUF R.2021.01 (Field no. LC17.01), adult male, collected by H.M. Ly on 25 August 2017 from Lung Cu Commune, Dong Van District, Ha Giang Province (23°21'N, 105°19'E, at an elevation of 1391 m a.s.l.). ***Paratypes*.**IEBR R.5151 (Field no. LC17.02), VNUF R.2021.03 (Field no. LC17.03), adult males, IEBR R.5152 (Field no. ST17.01), VNUF R.2021.08 (Field no. HG17.08), adult females, collected at the same locality as holotype on 25 August 2017; two adult females, VNUF R.2023.01 (Field no. LC22.01) and VNUF R.2023.02 (Field no. LC22.02), collected at the same locality as the holotype on 5 August 2022.

##### Diagnosis.

*Hemiphyllodactyluslungcuensis* sp. nov. can be distinguished from its congeners by a combination of the following characters: a maximum SVL of 44.2 mm; trunk not particularly elongate (AG/SVL ratio 0.52); chin scales 8–10, distinct enlarged postmentals; circumnasal scales two or three; supralabial scales 11 or 12; infralabial scales 10 or 11; ventral scale rows 6–9; dorsal scale rows 12–16; digital lamellae formula 4(3)–4–5(4)–4 (forefoot) and 4–5–5–5 (hindfoot); precloacal pores 17–25 and continuous series in males, 0–22 pitted precloacal scales in females; cloacal spur single in both sexes; enlarged subcaudal scales absent; dorsal surface brown sand color, with irregular dark brown streaks; a distinct dark postorbital stripe extending to at least the base of the neck, uneven dark streaks running along the flanks and ending at the base of tail, a cream-colored V-shaped postsacral mark with anteriorly projecting arms; a pale yellow ventral view of the body and limbs; and unpigmented caecum and gonads.

##### Description of the holotype.

Adult male, SVL 39.2 mm; tail length (TaL 37.3 mm); body compressed (TrunkL 20.3 mm); head triangular in dorsal profile, depressed, distinct from neck, and head longer than wide (HeadL 11.0 mm, HeadW 8.0 mm); eye large, pupils vertical (EyeD 1.8 mm); snout-eye distance (SnEye 4.3 mm); snout moderate, round in dorsal profile, longer than eye diameter (EyeD/SnEye 0.42); nare-eye distance (NarEye 3.2 mm); internarial distance (SnW 1.5 mm); ear opening oblique (EarD 0.7 mm).

***Proportions***: EarD/EyeD 0.39, TrunkL/SVL 0.52, HeadL/SVL 0.28, HeadW/SVL 0.20, HeadW/HeadL 0.73, SnEye/HeadL 0.39, NarEye/HeadL 0.29, EyeD/HeadL 0.16, SnW/HeadL 0.14, EyeD/NarEye 0.56, SnW/HeadW 0.19.

***Scalation***: Rostral large, wider than high, partially divided dorsally, bordered posteriorly by two large supranasals and two internasals (SnS); nares in contact with rostral anteriorly, first supralabial ventrally, supranasal dorsally, and three nasals posteriorly on each side; supralabial scales 12/12 (left/right), infralabial scales 11/10 (left/right), gradually decreasing in size towards angle of jaw; scales on head small, round, largest on rostrum; mental triangular, bordered by first infralabials and posteriorly by two enlarged postmentals, eight chin scales touching internal edge of infralabials and mental between the juncture of the second and third infralabials on each side of the head, anterior pair enlarged; ventral scales smooth and flat, much larger than dorsal scales, subimbricate, in seven rows (contained within one eye diameter); dorsal scales small, granular in 13 rows at midbody on dorsum (contained within one eye diameter), enlarged tubercles absent; fore-limbs relatively short, covered dorsally with granular, subimbricate scales, smaller smooth scales ventrally; 25 pore bearing femoral and precloacal sacles, in a continuous row; terminal two phalanges free, claws absent on first finger and on first toe,large triangular lamellae present on the second to fifth digits of both the forefoot and hindfoot pads, with digital formula 4–4–5–4 (forefoot) and 4–5–5–5 (hindfoot); four on first fingers, four on first toes; cloacal spurs (CloacS) one on each side; semi-regenerated tail; caudal scales flat, not forming distinct caudal segments; caecum and gonads unpigmented.

##### Coloration in life.

Dorsal surface of head, body, and limbs brown sand color, with irregularly shaped dark-brown streaks; a dark postorbital stripe, distinct, extending to at least base of neck; uneven dark streaks running along the flanks and ending at the base of tail; two dark dots on dorsal surface of tail; dorsal surface of tail has dark bands evenly spaced from base of tail to tip of tail; postsacral mark cream-colored and V-shaped, bearing anteriorly projecting arms; ventral surface of body and limbs pale yellow; subcaudal region pale peach.

##### Sexual dimorphism and variation.

The scale counts vary among the type series: chin scales 9 or 10; scales between supranasals one or three; supralabials 11; infralabials 10; ventral scale rows seven or nine; 0–22 pitted precloacal scales in females (distinct pore-bearing scales in males). Dorsal surface of the tail has dark bands evenly spaced from the base of the tail to the tip of the tail in females. Subcaudal region of original part of tail of the adult male paratype (IEBR R.5151) is reddish-orange, while the regenerated portion of the tail is uniformly dark grey. For other morphological characters of the paratypes are given in Table 8.

**Table 8.** Morphological characters of *Hemiphyllodactyluslungcuensis* sp. nov. (measurements in mm, * = regenerated or broken, ?= missing data, Min = minimum, Max = maximum, other abbreviations defined in the text).

	VNUF R.2021.01	IEBR R.5151	VNUF R.2021.03	VNUF R.2023.01	VNUF R.2023.02	IEBR R.5152	VNUF R.2021.08	Min-Max
	Holotype	Paratype	Paratype	Paratype	Paratype	Paratype	Paratype	
**Sex**	male	male	male	female	female	female	female	
**SVL**	39.2	43	44.2	35.3	37.4	39.2	43.5	35.3–44.2
**TaL**	37.3	25*	42.7	30.4*	27.3*	4.3*	34.7	34.7–42.7
**TrunkL**	20.3	23.0	23.1	17.0	19.3	19.0	22.3	17.0–23.1
**HeadL**	11.0	11.6	11.1	8.9	8.5	8.5	10.3	8.5–11.6
**HeadW**	8.0	8.0	7.0	6.9	7.0	7.3	8.0	6.9–8.0
**EyeD**	1.8	2.1	2.4	1.8	1.8	2.5	2.1	1.8–2.5
**SnEye**	4.3	4.5	5.0	3.6	3.9	4.1	4.2	3.6–5.0
**NarEye**	3.2	2.9	3.6	3.1	3.0	3.2	3.3	2.9–3.6
**SnW**	1.5	2.0	1.7	1.4	1.4	1.9	2.1	1.4–2.1
**EarD**	0.7	0.9	0.8	0.6	0.5	0.6	0.8	0.5–0.9
**EarD/EyeD**	0.39	0.43	0.33	0.33	0.28	0.24	0.38	0.24–0.43
**TrunkL/SVL**	0.52	0.53	0.52	0.48	0.52	0.48	0.51	0.48–0.53
**HeadL/SVL**	0.28	0.27	0.25	0.25	0.23	0.22	0.24	0.22–0.28
**HeadW/SVL**	0.20	0.19	0.16	0.20	0.19	0.19	0.18	0.16–0.20
**HeadW/HeadL**	0.73	0.69	0.63	0.78	0.82	0.86	0.78	0.63–0.86
**SnEye/HeadL**	0.39	0.39	0.45	0.40	0.46	0.48	0.41	0.39–0.48
**NarEye/HeadL**	0.29	0.25	0.32	0.35	0.35	0.38	0.32	0.25–0.38
**EyeD/HeadL**	0.16	0.18	0.22	0.20	0.21	0.29	0.20	0.21–0.29
**SnW/HeadL**	0.14	0.17	0.15	0.16	0.16	0.22	0.20	0.14–0.22
**EyeD/NarEye**	0.56	0.72	0.67	0.58	0.60	0.78	0.64	0.56–0.78
**SnW/HeadW**	0.19	0.25	0.24	0.20	0.20	0.26	0.26	0.19–0.26
**Chin**	8	8	?	10	10	9	10	8–10
**CN**	3	3	3	3	3	2	3	2–3
**SnS**	2	2	2	2	1	1	3	1–3
**SL (left/right)**	12/12	11/11	12/12	12/11	11/11	11/11	11/11	11–12
**IL (left/right)**	11/10	11/11	10/10	10/9	10/9	10/10	10/10	9–11
**VS**	7	6	7	10	11	9	7	6–11
**DS**	13	13	15	17	16	16	12	12–17
**Lamellae formula of forelimbs II–V (left)**	4454	*	44??	4454	4454	4444	3454	4(3)45(4)4
**Lamellae formula of hind limbs II–V (left)**	4555	4555	4555	4555	4555	4555	4555	4555
**SL1F**	4	4	?	4	4	4	3	3–4
**SL1T**	4	4	4	4	4	4	4	4
**Total femoroprecloacal pores**	25	17	23	16	0	0	22	17–25 (in males)
**CloacS on each side**	1	1	1	2	1	1	1	1–2
**Precloacal and femoral pore series separate (1) or continuous (0)**	0	0	0	0			0	0
**Subcaudals enlarged, plate-like**	0	0	0	0	0	0	0	0

##### Distribution.

*Hemiphyllodactyluslungcuensis* sp. nov. is currently known only from the type locality in Lung Cu Commune, Dong Van District, Ha Giang Province, northeastern Vietnam (Fig. 1).

##### Etymology.

Specific epithet *lungcuensis* is a toponym in reference to the type locality of the species. For the common names, we suggest Lungcu Slender Gecko (English) and Thạch sùng dẹp lũng cú (Vietnamese).

##### Natural history.

**Figure 9.**
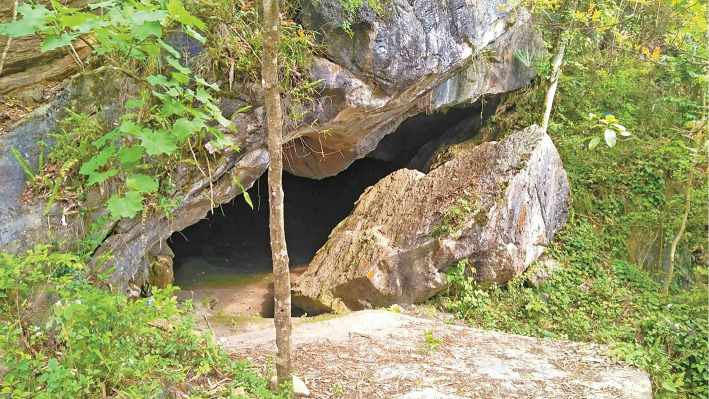
Habitat of *Hemiphyllodactyluslungcuensis* sp. nov. in Lung Cu Commune, Dong Van District, Ha Giang Province, northeastern Vietnam.

The species is known from a karstic habitat. The type series were found on karst cliffs between 19:00 and 20:30, approximately 1–1.5 m above the ground. The surrounding habitat was a disturbed karst forest with small hardwood and shrub trees, and was located near residential areas and road systems (Fig. 9). The type locality of the new species is located in an unprotected area, and thus we need to clarify the impact and threats of the localized disturbance so as to put in place effective conservation measures.

##### Comparisons.

The molecular analyses indicate that *Hemiphyllodactyluslungcuensis* sp. nov. is a phylogenetically distinct lineage of the clade 6 that is not embedded within any lineage of the other 13 species of clade 6 (Agung et al. 2022) and bears an uncorrected pairwise sequence divergence of 4.6–20.2% from them (Fig. 1, Table 2). *Hemiphyllodactyluslungcuensis* sp. nov. is the sister species to a clade composed of *H.huishuiensis* and *H.yanshanensis* (Fig. 1) from which it bears an uncorrected pairwise sequence divergence of 5.3% from *H.huishuiensis* and 6.9% from *H.yanshanensis* (Table 2). *Hemiphyllodactyluslungcuensis* sp. nov. differs significantly from *H.huishuiensis* in mean values of EyeD (0.31 vs. 0.41, p = 0.001) and SnW (0.23 vs. 0.08, p = 0.001) and from *H.yanshanensis* in mean values of TrunkL (1.31 vs. 1.29, p = 0.03) and EyeD (0.31 vs. 0.38, p = 0.002). In addition, *Hemiphyllodactyluslungcuensis* sp. nov. differs significantly from *H.nahangensis* in mean values of EyeD (0.31 vs. 0.42, p = 0.001), SnW (0.23 vs. 0.05, p = 0.001), DS (14.75 vs. 21, p = 0.001), and SL1T (4 vs. 3, p = 0.001) (Tables 6, 7). Statistically significant differences of morphometric and meristic traits between *Hemiphyllodactyluslungcuensis* sp. nov. and all other species in clade 6 are presented in Tables 6, 7. Due to the unavailability of individual specimens of *H.dushanensis* for statistical analysis, we obtained integrated data of this species from Do et al. (2020: table 4) for comparison, the new species can be distinguished from *H.dushanensis* by the presence of dark postorbital stripe, dark dorsolateral stripe, and dark dorsal transverse blotches (vs. absent in *H.dushanensis*).

## ﻿Discussion

Phylogenetic analyses indicate that the newly discovered species is closely related to *H.nahangensis* (with a genetic pairwise distance of 4.6%) found in Vietnam and *H.dushanensis* (with a genetic pairwise distance of 4.8%) found in China. However, the type locality of the new species is located in Lung Cu Commune, Dong Van District, Ha Giang Province, northeastern Vietnam, which is approximately 100 km away from the type locality of *H.nahangensis* in Sinh Long Commune, Na Hang District, Tuyen Quang Province, northeastern Vietnam (Do et al. 2020) and approximately 350 km away from the type locality of *H.dushanensis* in Dushan County, Guizhou Province, China. As such, there is significant geographical separation between the new species and its closest known relatives.

The recognition of *Hemiphyllodactyluslungcuensis* sp. nov. increases the number of *Hemiphyllodactylus* in Vietnam to eight. However, the diversity of *Hemiphyllodactylus* in Vietnam is still likely underestimated, particularly in karst landscapes in northern Vietnam (Do et al. 2020). In comparison, the southern Chinese provinces of Yunnan and Guangxi, which run along the border with Vietnam, have seen the description of three new species of *Hemiphyllodactylus* from the karst forests in the last two years (Agung et al. 2021, 2022; Uetz et al. 2022). Therefore, further field research is required to uncover the unrealized diversity of *Hemiphyllodactylus* in the largest karst formation in northern Vietnam.

## Supplementary Material

XML Treatment for Hemiphyllodactylus lungcuensis

## ﻿Additional information

### Author ORCIDs

Vinh Quang Luu https://orcid.org/0000-0002-0634-1338

Thuong Huyen Nguyen https://orcid.org/0000-0002-4303-525X

Quyen Hanh Do https://orcid.org/0000-0002-9437-4673

Cuong The Pham https://orcid.org/0000-0001-5158-4526

Truong Quang Nguyen https://orcid.org/0000-0002-6601-0880

Minh Duc Le https://orcid.org/0000-0002-2953-2815

Thomas Ziegler https://orcid.org/0000-0002-4797-609X

Jesse L. Grismer https://orcid.org/0000-0002-2542-5430

L. Lee Grismer https://orcid.org/0000-0001-8422-3698

### Data availability

All of the data that support the findings of this study are available in the main text or Supplementary Information.
